# Influence of Different Selenium Biofortification Methods on Structural Features and Antioxidant Bioactivities of *Pleurotus geesteranus* Polysaccharides

**DOI:** 10.3390/foods15101660

**Published:** 2026-05-09

**Authors:** Lingyang Yao, Zhengyu Bao, Huan Tian, Tao Feng, Min Sun, Yuanting Liang, Lingyun Yao, Hui Ma

**Affiliations:** 1 Faculty of Materials Science and Chemistry, China University of Geosciences, Wuhan 430074, China; qyangw@outlook.com (L.Y.); zybao@cug.edu.cn (Z.B.); huantian@cug.edu.cn (H.T.); 2 Faculty of Flavour Fragrance and Cosmetics, Shanghai Institute of Technology, Shanghai 201418, China; fengtao@sit.edu.cn (T.F.); sunmin@sit.edu.cn (M.S.); soyjle_2000@163.com (Y.L.); m1061382063@163.com (H.M.)

**Keywords:** selenium-enriched polysaccharides, ultrasound-assisted extraction, monosaccharide composition, antioxidant bioactivity

## Abstract

Selenium (Se) biofortification is considered an effective approach to enhance the nutritional and functional properties of fungal polysaccharides. In this study, *Pleurotus geesteranus* was biofortified with different Se sources including sodium selenite [Se(IV)], sodium selenate [Se(VI)], potassium 2-selenocyanatoacetate (PSeCA), and selenium ore powder (SeOP) to obtain Se-enriched polysaccharides by ultrasound-assisted extraction (UAE). UAE parameters were optimized via a Box–Behnken design; the optimized UAE model exhibited high predictability (R^2^ = 0.9886, CV = 1.71%), enabling reliable scale-up for industrial extraction, with the PSeCA group achieving the highest polysaccharide content (75.12 mg/g) and Se level (13.85 mg/kg) compared to the control and other Se-fortified groups. The monosaccharide composition analysis on polysaccharides revealed that Se(IV) and Se(VI) primarily increased mannose and fructose contents, whereas PSeCA and SeOP exhibited characteristic glucose-dominant profiles. Furthermore, the molecular weight (Mw) distribution of fungal polysaccharides could be altered under biofortification conditions. In addition, polysaccharides of each group revealed different antioxidant bioactivities among tested free radicals. The result indicated that PSeCA as an organic Se source, rarely studied, has promising potential in *P. geesteranus* biofortification for obtaining antioxidant Se-polysaccharides.

## 1. Introduction

Mushrooms are widely recognized for their nutritional and medicinal value, in which polysaccharides constitute the most prominent bioactive components [1,2]. These macromolecules exert diverse physiological effects, including antitumor, immunomodulatory, anti-inflammatory, hypoglycemic, hypolipidemic, antioxidant, and antiviral activities [3,4,5]. Due to their broad bioactive capacity and therapeutic potential, applications for mushroom polysaccharides have been extensively explored in the fields of functional foods, nutraceuticals, and pharmaceuticals [6,7]. Moreover, the development and application of selenium (Se)-enriched polysaccharides of mushroom have attracted increasing attention in recent years [8,9,10].

The biofortification of mushroom via exogenous Se species is an important way to derive Se-polysaccharides, because fungi have a strong capacity to bioaccumulate Se and convert it into organic Se forms including Se-containing polysaccharides [11,12]. It has been reported that about 20% of Se can combine with polysaccharides in *Calocybe indica* and *Flammulina velutipes* using the biofortification process [13,14]. Additionally, Se-containing polysaccharides could be impacted by exogenous Se species during the biofortification process. For example, selenium yeast and sodium selenite biofortified *Pleurotus eryngii* have a lower molecular weight of polysaccharides compared to the non-biofortified control, with the same monosaccharides observed in varying molar ratios [9].

*Pleurotus* species are commercially important edible mushrooms cultivated globally; several studies have focused on obtaining mushroom-derived Se-proteins and Se-polysaccharides through the biofortification of *Pleurotus* species through organic and inorganic Se sources [9,15,16]. In a recent work, *P. geesteranus* exhibited efficient Se enrichment capacity using the biofortification process, with high Se content (6.53–10.25 mg/kg) detected in fruiting bodies when biofortified with various exogenous Se species including selenite [Se(IV)], selenate [Se(VI)], potassium,2-selenocyanatoacetate (PSeCA), and selenium ore powder (SeOP) [17]. More importantly, selenoamino acids could be greatly changed when fortified with these four Se species [17]. Due to the highest Se content (10.25 mg/kg) observed in PSeCA-fortified *P. geesteranus*, we hypothesized that organic Se might integrate more efficiently into polysaccharide structures than inorganic forms, resulting in higher Se content and enhanced antioxidant activity. However, the effect of Se species on the monosaccharide composition and molecular weight distribution of *P. geesteranus* polysaccharides remains unexplored.

Se(IV) and Se(VI) are the principally used Se sources for mushroom biofortification, while it generally responds better to organic Se (such as PSeCA) on fungus biomass and Se-metabolite accumulation [11]. Additionally, SeOP represents a slow-release natural mineral form, allowing comparison of bioavailability effects on polysaccharide synthesis [18]. Based on the biofortification methods developed in *P. geesteranus* cultivation [17], the primary objective of this study was to evaluate the impacts of four exogenous Se species (i.e., Se(IV), Se(VI), PSeCA, and SeOP) on Se-polysaccharide bioaccumulation in fruiting bodies. Ultrasound-assisted extraction (UAE) was used to recover polysaccharides from fruiting bodies of *P. geesteranus* after optimization by the response surface design method. Additionally, different Se sources can influence the structure and property of Se-polysaccharides using the fortification process. Therefore, another objective of this study was to explore the impact of the four Se species on the structure and antioxidant bioactivity of mushroom-derived polysaccharides. While previous studies focused solely on selenite or selenate, the four Se species compared here reveal distinct metabolic fates and offer nuanced guidance for selecting a biofortification method based on desired polysaccharide functionality.

## 2. Materials and Methods

### 2.1. Reagents and Chemicals

Common reagents including phenol, ethanol, methanol, sulfuric acid, hydrochloric acid, sodium hydroxide, and potassium bromide were acquired from Sinopharm Chemical Reagent Co., Ltd. (Shanghai, China). Se species including Se(IV) and Se(VI) with purity of over 98% were purchased from Sigma-Aldrich (Shanghai, China), SeOP (Se content of 80 mg/kg) was supplied by Shaanxi Ziyang Zhongdida Se-Technology Co., Ltd. (Ankang, China), and PSeCA (purity > 99%) was provided by Hubei Zhongdi Xineng Technology Co., Ltd. (Wuhan, China). Standards including Se(VI), bovine serum albumin (BSA) and glucose were all of high purity (>98%) and purchased from Sigma-Aldrich (Shanghai, China). All other chemicals and reagents used in this study were of analytical grade and purchased from Sigma-Aldrich (Shanghai, China).

### 2.2. Preparation of P. geesteranus Fruiting Body Samples

The strain *P. geesteranus* Jinxiu was used for sample preparation and the cultivation of mushrooms was conducted according to the method described previously [17]. Firstly, liquid inoculum was prepared using 80 mL of potato dextrose broth (PDB) inoculated with a loop of strain and shake cultured at 25 °C for 7 days. Subsequently, the liquid inoculum (20 mL) was inoculated in a polypropylene bag filled with 1.4 kg of the substrate and incubated in the dark at 25 °C for 17 days of spawn running. The spawn running substrate comprised corncob 380 g/kg, cottonseed shell 300 g/kg, bran 200 g/kg, wood chips 110 g/kg, soybean meal 6 g/kg, lime 2 g/kg, and calcium bicarbonate 2 g/kg with pH adjusted to 8.5 and the moisture content maintained at about 70% [17]. After 17 days of spawn running, the fully colonized bags were kept at 25 °C and 80% relative humidity for vegetative (32 days) and subsequent fruiting body (6 days) cultivation. Four Se biofortification methods were established by supplementing different Se species in the non-seleniferous substrate for spawn preparation and subsequent mushroom cultivation, with the content of Se(IV) at 2000 μg/kg, Se(VI) at 1000 μg/kg, PSeCA at 2000 μg/kg, and SeOP at 2000 μg/kg. The non-Se-treated bags were also inoculated for fruiting body harvesting and used as blank control (CK). Mushroom cultivation experiments were performed in at least triplicates, and fruiting bodies were collected at terminal phase of maturation. All harvested fruiting bodies were freeze-dried, ground into fine powder, and stored at 4 °C until use.

### 2.3. Ultrasound-Assisted Extraction (UAE) Parameter Optimization

#### 2.3.1. Single Factor Experiments

For polysaccharide extraction, freeze-dried mushroom samples were firstly powdered and mixed with 95% ethanol (7 mL/g) to remove lipid-soluble impurities. The defatted mushroom residue was dried at 60 °C to a constant weight in an oven. Subsequently, the defatted powder was dispersed in deionized water at a predetermined solid-to-liquid ratio for polysaccharide extraction, with the impact of UAE parameters on extraction yield assessed by single-factor experiments. Extraction procedures were performed with ultrasonic power ranging from 100 W to 500 W, extraction time ranging from 10 min to 50 min, temperature ranging from 50 °C to 90 °C, and liquid–solid ratio ranging from 10 mL/g to 30 mL/g (Figure S1). After UAE, the mixture was allowed to cool naturally to room temperature, followed by filtration through cheesecloth and vacuum filtration to obtain the crude extract. The resulting filtrate was collected for subsequent concentration, deproteinization, and ethanol precipitation steps [19].

#### 2.3.2. Box–Behnken Design for Extraction Optimization

Based on the results of single-factor experiments, a three-level Box–Behnken design (BBD) combined with response surface methodology (RSM) was employed to further optimize UAE parameters [19]. The independent variables included ultrasonic power (200, 300, and 400 W), ultrasonic duration (20, 30, and 40 min), extraction temperature (70, 80, and 90 °C), and liquid-to-solid ratio (15:1, 20:1, and 25:1 mL/g). Total polysaccharide yield was selected as the response variable. The BBD comprised 29 experimental runs, with optimal extraction parameters identified by comparing polysaccharide yields across all runs. To elucidate the quantitative relationships between the response and the independent variables, the experimental data were fitted to a second-order polynomial equation, and a quadratic response surface model was subsequently established to assess the effects of each factor and their interactions on polysaccharide yield:

$$Y=\beta_{0}+\sum \beta_{i}X_{i}+\sum \beta_{ii}X_{i}^{2}+\sum \beta_{ij}X_{i}X_{j}$$

where *Y* represents the predicted polysaccharide extraction yield, $\beta_{0}$ is the regression coefficient of the intercept, *β_i_* denotes the regression coefficients of the linear terms, *β_ii_* represents the regression coefficients of the quadratic terms, *β_ij_* corresponds to the regression coefficients of the interaction terms, and *X_i_* and *X_j_* are the independent variables.

### 2.4. Total Sugar Content Determination and Polysaccharide Purification

The total sugar contents of each extraction were determined by the phenol–sulfuric acid method with D-glucose used as standard [19]. Briefly, 1 mL of the sample solution was placed in a colorimetric tube, followed by the addition of 1 mL of 5% (*w*/*v*) phenol solution. Then, 5 mL of concentrated sulfuric acid was rapidly added along the tube wall, mixed thoroughly, and allowed to stand at room temperature for 30 min for color development. Glucose was used as the standard to prepare a series of standard solutions (0–100 μg/mL), which were treated under the same conditions to construct a calibration curve. The absorbance of each sample was measured at 490 nm using a UV–Vis spectrophotometer (Unico Shanghai Instrument Co., Ltd., Shanghai, China), and the polysaccharide concentration was calculated based on the glucose standard curve.

For polysaccharide purification, the extracted crude polysaccharide solutions of *P. geesteranus* were firstly treated with 8% papain (pH 5.5) and incubated at 50 °C for 2.5 h to hydrolyze contained proteins [19]. Subsequently, the solution was heated to 100 °C for 10 min and centrifugated at 4000× *g* for 30 min to remove the precipitation (papain), and the supernatant solution was added with four volumes of pure ethanol and kept overnight at 4 °C to obtain the deproteinated polysaccharide precipitation. Thereafter, the deproteinated polysaccharides were further purified by DEAE Sepharose Fast Flow column chromatography (3.5 cm × 30 cm) method [20]. Specifically, the column was eluted with 0, 0.1, 0.3, and 0.5 M NaCl aqueous solutions of 300 mL each at 1 mL/min, with fraction collection volume of 10 mL per tube. Thereafter, each collected elution (10 mL/tube) was gathered based on their total sugar contents using phenol–sulfuric acid assays, and the gathered fraction (eluted with 0.1 M NaCl) was dialyzed to remove small molecules and freeze-dried for further use.

### 2.5. Total Se Content Analysis of P. geesteranus Polysaccharides

The total Se content of extracted polysaccharides was determined by atomic fluorescence spectrometry (AFS), reported previously with slight modification [17]. Briefly, approximately 0.1 g of freeze-dried polysaccharide sample was placed in a digestion vessel and mixed with 2 mL of concentrated HNO_3_. The mixture was digested at 190 °C for 30 min using a Mars 6 microwave digestion system (CEM Corporation, Matthews, NC, USA). After cooling, the digest was evaporated to near dryness on a hot plate (120 °C) and then treated with 1 mL of 6 mol/L HCl to convert Se into the Se(IV) oxidation state. The solution was subsequently diluted to 10 mL with ultrapure water. The final solution was introduced into the atomizer through a hydride generation unit with an argon carrier gas, and the total Se concentration was determined using an AFS-9230 atomic fluorescence spectrometer (Jitian Instruments Co., Ltd., Beijing, China).

### 2.6. Physicochemical Characterization of P. geesteranus Polysaccharides

#### 2.6.1. Monosaccharide Composition and Molecular Weight Analysis

The monosaccharide composition of each purified polysaccharide sample was determined by an ion chromatography (Thermo Fisher ICS5000 System, Waltham, MA, USA) method [20]. Each purified polysaccharide sample (10 mg) was thoroughly hydrolyzed by 1 mL of 2.5 M trifluoroacetic acid (TFA) at 121 °C for 2 h, and the TFA was removed with nitrogen and methanol. Subsequently, each hydrolyzed sample (5 μL) was separated by a Dionex Carbopac™ PA20 column (3 mm × 150 mm); the chromatography was performed at column temperature of 30 °C and flow rate of 0.5 mL/min. Monosaccharides were identified by comparing retention times and peak areas with authentic standards.

The molecular weight distribution of each polysaccharide sample was measured using high-performance gel permeation chromatography (HPGPC) using Agilent 1260 Infinity system (Santa Clara, CA, USA) coupled with a Waters Ultrahydrogel column (7.8 mm × 300 mm, Tosoh Corp., Tokyo, Japan) and an ELSD-UM 5800 Plus detector (Unimicro Technologies Co., Ltd., Shanghai, China). Briefly, each purified polysaccharide sample solution (8 mg/mL) was firstly filtered through a 0.22 μm membrane, and injected at a volume of 20 μL for HPGPC analysis. The mobile phase consisted of 0.1 mol/L NaNO_3_ with a flow rate of 0.5 mL/min. The calibration curve was obtained by using various dextran standards with molecular weight ranging from 4320 to 200,000 Da, and the molecular weight parameters (Mn, Mw, Mw/Mn) were calculated from the constructed calibration curve [20].

#### 2.6.2. Fourier Transform–Infrared (FT-IR) Spectroscopy Analysis

Purified polysaccharide samples were characterized by Fourier transform–infrared (FT-IR) spectroscopy to investigate their functional groups and structural features [20]. The dried polysaccharide samples were thoroughly mixed with spectroscopic grade anhydrous potassium bromide (KBr) at a mass ratio of 1:10 (*w*/*w*) and ground uniformly in a mortar. The mixture was then pressed into transparent pellets. FT-IR spectra were recorded on a Nicolet iS50 FT-IR spectrometer (Thermo Fisher Scientific, USA) over a scanning range of 4000–500 cm^−1^, and the resulting spectra were used for structural analysis of the polysaccharides.

#### 2.6.3. Nuclear Magnetic Resonance (NMR) Spectroscopy

Structural characterization of purified polysaccharides was performed using ^1^H and ^13^C NMR on a Bruker AVANCE III HD 400 MHz spectrometer (9.4 T). Lyophilized polysaccharide samples (15–20 mg) were dissolved in 0.5 mL D_2_O, sonicated, and centrifuged. The supernatant was transferred into a 5 mm NMR tube. ^1^H NMR spectra were acquired at 25 °C using a standard zg pulse program (32 scans), and ^13^C spectra using the zgpg30 sequence (2000–5000 scans). Spectral processing and peak assignments were performed in MestReNova by referencing the literature and database values.

#### 2.6.4. Scanning Electron Microscopy (SEM) Analysis

The surface morphology of the polysaccharides was examined by SEM following the previously reported method with slight modification [20]. Briefly, dried polysaccharide samples were evenly dispersed onto double-sided conductive carbon tape mounted on aluminum stubs and subsequently sputter-coated with a thin layer of gold to enhance surface conductivity and imaging quality. SEM observations were performed using a field-emission scanning electron microscope (FE-SEM, Hitachi SU8010, Hitachi Ltd., Tokyo, Japan). Images were acquired at various magnifications at an accelerating voltage of 20 kV, a working distance of approximately 10 mm, and an appropriate beam current to ensure optimal resolution. The obtained micrographs were used to characterize the microstructural and morphological features of tested samples.

### 2.7. Antioxidant Activity Determination

#### 2.7.1. DPPH Scavenging Assay

The antioxidant activity of polysaccharide extracts was evaluated using the DPPH radical scavenging assay following the method described previously with minor modification [19]. Firstly, the dried polysaccharide sample was serially diluted to appropriate concentrations. Then, 1.0 mL of the sample solution was mixed with 1.0 mL of 80 μg/mL DPPH solution prepared in 70% ethanol. The mixture was incubated in the dark at room temperature for 30 min. After incubation, the absorbance of each mixture was measured at 517 nm. The DPPH radical scavenging activity was calculated by the equation DPPH Inhibition (%) = [1 − (*A*_1_ − *A*_2_)/*A*_0_] × 100%, where *A*_0_ represents the absorbance of the control solution containing DPPH working solution and 70% ethanol (maximum absorbance), *A*_1_ represents the absorbance of the reaction mixture containing the sample solution and DPPH working solution, and *A*_2_ represents the absorbance of the sample blank containing the sample solution and 70% ethanol (to correct for background absorbance).

#### 2.7.2. ABTS^+^ Scavenging Assay

The ABTS radical scavenging activity of each sample was determined according to a modified method previously described [19]. Briefly, equal volumes of 7 mmol/L ABTS solution and 2.45 mmol/L potassium persulfate (K_2_S_2_O_8_) solution were mixed and incubated in the dark at room temperature for 24 h to generate the ABTS^+^ radical cation. The resulting ABTS^+^ working solution was then diluted with 70% ethanol to obtain an absorbance of 0.700 ± 0.10 at 734 nm. The polysaccharide sample was serially diluted to appropriate concentrations (40 μL) and mixed with diluted ABTS^+^ working solution (160 μL), and the mixture was incubated in the dark at room temperature for 10 min to record the absorbance at 734 nm. The ABTS radical scavenging activity was calculated: ABTS^+^ Inhibition (%) = [1 − (*A*_1_ − *A*_2_)/*A*_0_] × 100%, where *A*_0_ represents the absorbance of the control mixture containing 40 μL of 70% ethanol and 160 μL of the ABTS^+^ working solution, *A*_1_ represents the absorbance of the reaction mixture containing 40 μL of the sample solution and 160 μL of the ABTS^+^ working solution, and *A*_2_ represents the absorbance of the sample blank containing 40 μL of the sample solution and 160 μL of 70% ethanol.

#### 2.7.3. Hydroxyl Radical (·OH) Scavenging Assay

The ·OH scavenging activity of the polysaccharide sample was determined using the Fenton reaction-based method described previously [19]. In brief, each polysaccharide sample was firstly dissolved in deionized water to appropriate concentrations. Then, 50 μL of the sample solution, 50 μL of 6 mmol/L H_2_O_2_ solution, 50 μL of 6 mmol/L salicylic acid solution, and 50 μL of 6 mmol/L FeSO_4_ solution were sequentially added into a 96-well microplate. The reaction mixture was incubated at room temperature in the dark for 15 min, and the absorbance was measured at 510 nm. Thereafter, the radical scavenging rate was calculated according to the equation ·OH Inhibition (%) = [1 − (*A*_1_ − *A*_2_)/*A*_0_] × 100%, where *A*_0_ represents the absorbance of blank control that uses 50 μL deionized water instead of sample solution, *A*_1_ represents the absorbance of the reaction mixture containing 50 μL of the sample solution, and *A*_2_ represents the absorbance of the control that uses 50 μL deionized water instead of H_2_O_2_ solution.

### 2.8. Statistical Analysis

All experiments were conducted in triplicate, and the results were expressed as mean ± standard deviation (SD). One-way analysis of variance (ANOVA) followed by Duncan’s multiple range test was applied to determine significant differences among means. Statistical analyses were performed using IBM SPSS Statistics 29.0.1.0 (IBM Corp., Armonk, NY, USA), and mean values were considered significantly different at *p* < 0.05. For optimization experiments, the response surface methodology (RSM) based on the Box–Behnken design was carried out using Design-Expert software (Version 13, Stat-Ease Inc., Minneapolis, MN, USA).

## 3. Results and Discussion

### 3.1. Optimization of Polysaccharide Extraction by RSM

The optimal conditions for polysaccharide extraction based on single-factor experiments were ultrasonic power of 300 W, extraction time of 30 min, extraction temperature of 80 °C, and a liquid-to-material ratio of 20 mL/g (Figure S1). These findings align with previous studies on extracting polysaccharides from *P. ostreatus* mushroom by the UAE method [21]. In addition, an RSM design was employed in order to further optimize the UAE parameters, with a total of 29 runs carried out. The results are presented in Table 1. As shown in Table 1, the polysaccharide extraction yield ranged from 46.39 mg/g to 69.81 mg/g. Based on the experimental data, a multiple regression fitting was performed, yielding the following second-order polynomial regression equation for polysaccharide yield: Y = 68.94 + 2.65A + 0.9567B − 4.77C + 0.8542D − 0.3425AB − 0.7125AC − 0.2575AD + 0.0250BC + 0.0225BD − 0.1225CD − 8.40A^2^ − 3.94B^2^ − 9.20C^2^ − 2.40D^2^. The predicted maximum polysaccharide extraction yield was 70.74 mg/g.

The analysis of variance (ANOVA) for the regression model is shown in Table 2. The results indicated that the model was highly significant (*p* < 0.001), with an adjusted *R*^2^ value close to 1, demonstrating a high goodness of fit and the model’s ability to reflect the trends in the experimental data. The coefficients of variation (CV) for the response variables were all less than 10%, indicating that the experimental data possessed good precision and reproducibility. Moreover, the non-significant lack-of-fit test (*p* > 0.05) confirmed that the model was free from systematic bias and could adequately explain the variability in the response variables [22]. These results validate the robustness and predictive capacity of the regression model, confirming its suitability to optimize the extraction process.

**Figure 1:**
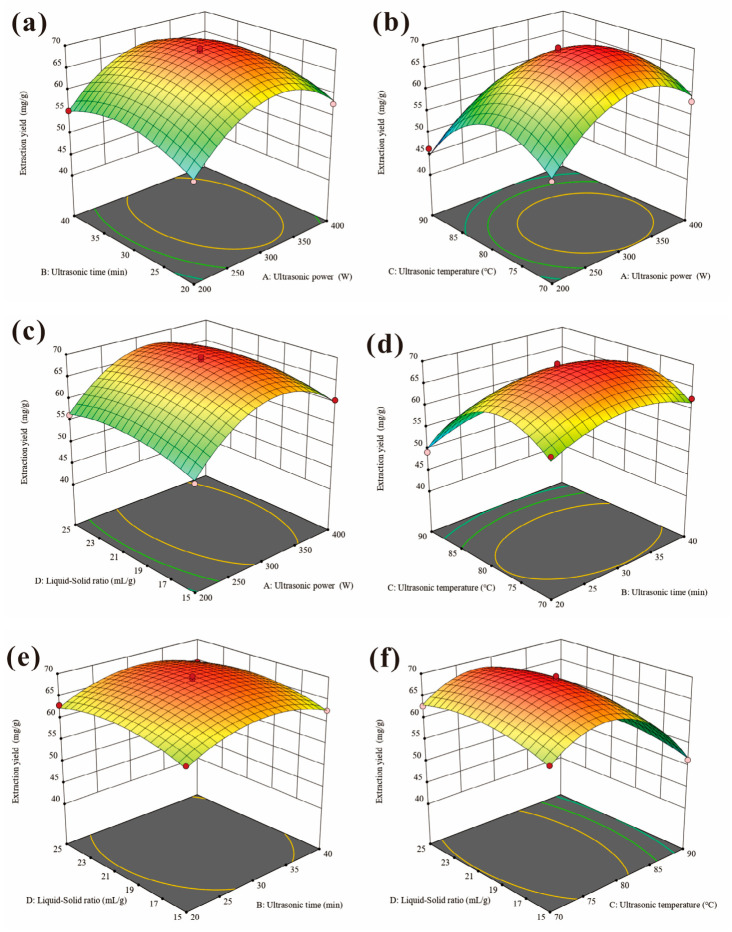
Three-dimensional response surfaces for the effects of two various factors on the polysaccharide extraction yield. (**a**) Ultrasonic power and extraction time, (**b**) ultrasonic power and extraction temperature, (**c**) ultrasonic power and liquid-to-solid ratio, (**d**) extraction temperature and extraction time, (**e**) liquid-to-solid ratio and extraction time, (**f**) liquid-to-solid ratio and extraction temperature.

The interaction effects between ultrasonic power (A), ultrasonic time (B), ultrasonic temperature (C), and the liquid-to-solid ratio (D) on polysaccharide extraction yield are illustrated in Figure 1. The results revealed that all factors exhibited significant quadratic interaction effects, with the response surface showing an upward convex trend in the central region (Figure 1). This suggested that the extraction yield increased with the enhancement of process parameters within an optimal range, while decreasing once the parameters exceed the optimal limits. For example, increased ultrasonic power and extraction time enhanced the extraction yield initially, while its value decreased when further increasing the power and extraction time (Figure 1a). As reported elsewhere, prolonged treatment time and excessive power might damage the polysaccharide structure and cause polysaccharide chain degradation, leading to a decrease in yield [23]. Commonly, the extraction performance is jointly governed by the balance between energy input, mass transfer dynamics, and the structural stability of polysaccharides [24]. Under the optimized conditions of ultrasonic power 320 W, extraction time 31 min, extraction temperature 77 °C, and a liquid-to-solid ratio of 21 mL/g, the experimental polysaccharide yield reached 70.26 mg/g. This value showed excellent agreement with the predicted yield of 69.88 mg/g, thereby demonstrating the high accuracy, robustness, and predictive reliability of the established model. Additionally, the optimized UAE method improves extraction time and solvent consumption compared to conventional hot water extraction, aligning with green chemistry principles for sustainable functional food production.

### 3.2. Se Content of Polysaccharides Derived from Different Mushroom Samples

Polysaccharides of the four Se-biofortified mushroom samples were extracted based on the optimized UAE parameters, with the extraction yield and Se content of polysaccharides detected [4]. As shown in Table 3, Se-biofortified samples resulted in similar polysaccharide yields (*p* > 0.05) compared to the control group (70.26 mg/g). Nevertheless, PSeCA-fortified mushrooms achieved the highest polysaccharide yield (75.12 mg/g) with a relative (*p* > 0.05) increase in polysaccharide accumulation tendency (Table 3). The result suggested PSeCA revealed a potential capacity to modulate fungal metabolic activity and promote polysaccharide biosynthesis. This observation is consistent with previous findings indicating that organic Se forms are more readily incorporated into and regulate fungal metabolic pathways [25]. Additionally, organic Se sources are usually more efficiently incorporated into cellular metabolism and stimulate polysaccharide synthesis under relatively low cytotoxic stress [11]. In contrast, Se(VI) exhibited a relative inhibition effect on polysaccharide accumulation (Table 3). It is well known that high levels of inorganic Se must undergo reductive transformation and complex biochemical processing, which might consume intracellular reducing power, trigger oxidative stress, and ultimately constrain the polysaccharide biosynthesis [26].

The Se content of polysaccharides was determined by AFS after chromatography purification. The result revealed the Se content of polysaccharide significantly (*p* < 0.05) increased, suggesting Se-polysaccharide could be formed using the fortification process (Table 3). The highest Se content was found in the PSeCA group (13.85 mg/kg), which was approximately 38.5 times that of the control group. This was followed by Se(VI) (8.34 mg/kg), SeOP (7.93 mg/kg), and Se(IV) (5.57 mg/kg). These results suggested the following order of Se-polysaccharide bioaccumulation efficiency: PSeCA > Se(VI) > SeOP > Se(IV) > CK. This trend is consistent with the reported pattern of Se absorption efficiency in the biofortification process [27]. The polysaccharide yield and Se content results indicated that PSeCA exhibited the highest polysaccharide production and excellent Se bioaccumulation efficiency.

### 3.3. Monosaccharide Composition and Molecular Weight of Polysaccharides

**Figure 2:**
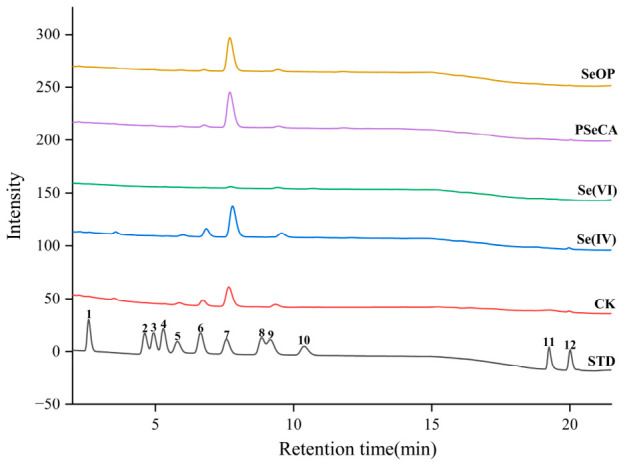
Monosaccharide composition of *P. geesteranus* polysaccharides by HPLC. Peaks correspond to: (1) fucose, (2) amino-galactose, (3) rhamnose, (4) arabinose, (5) amino-glucose, (6) galactose, (7) glucose, (8) xylose, (9) mannose, (10) fructose, (11) galacturonic acid, (12) glucuronic acid.

The mushroom polysaccharides were primarily composed of several neutral monosaccharides and a small number of acidic sugars, with glucose as the dominant sugar, indicating that the polysaccharides mainly consist of glucan (Figure 2 and Table S1). This result is consistent with the typical polysaccharide structure found in edible fungi [28,29]. Typically, glucan (β-1→3/1→6-glucan) is the primary immunomodulatory component in edible mushroom polysaccharides [9,30]. The glucose accounted for 65.25% of total sugar contents in the control group (CK), while mannose and galactose accounted for 9.36% and 15.90% respectively (Table S1). However, the monosaccharide composition of *P. geesteranus* polysaccharides was significantly influenced by exogenous Se species. Specifically, the Se(VI) treatment significantly reduced the glucose content (38.55%) while increasing the proportions of mannose (39.41%) and fructose (17.69%). The altered monosaccharide profile suggests possible metabolic pathway shifts, which require further investigation. The increase in mannose content is typically associated with an enhanced mannan structure in fungal cell walls, which may further improve intestinal adhesion and immune receptor recognition [29,31]. In contrast, the PSeCA and SeOP treatment groups showed significantly higher glucose contents (85.82% and 88.69% respectively) as compared to the control group (Table S1). The result indicated that glucose might be more favored as a backbone in polysaccharide biosynthesis when biofortified with PSeCA and SeOP. Additionally, small amounts of acidic sugars, such as galacturonic acid and glucuronic acid, were detected in the Se(VI), PSeCA, and SeOP treatment groups, suggesting that Se treatment induced an increase in acidic groups in the fungal cell walls. The elevated acidic sugar content under Se biofortification might enhance the antioxidant capacity of the mushroom polysaccharides [27]. Consequently, variations in monosaccharide profiles suggested that Se treatment might modulate biosynthetic pathways of *P. geesteranus* and influence the structural characteristics and bioactivity of fungal polysaccharides.

**Figure 3:**
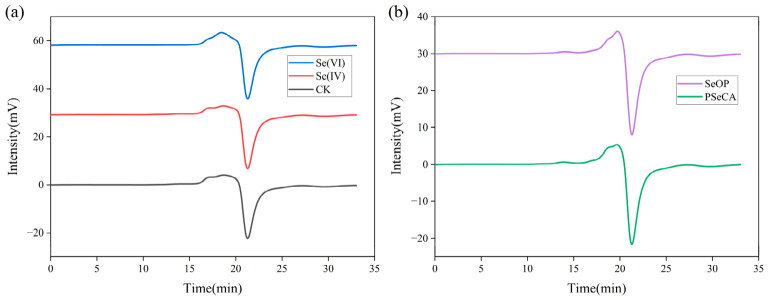
Molecular weight distribution of *P. geesteranus* polysaccharides. (**a**) Control check (CK), Se(IV) treatment, and Se(VI) treatment; (**b**) PSeCA treatment, and SeOP treatment.

The Mw of each polysaccharide sample was further determined by HCGPC to test whether Se biofortification induces depolymerization or aggregation of glucan chains, which could directly impact immunomodulatory activity [9]. The standard curve for the polysaccharides was *Y* = −0.5558*X* + 13.8267, with a high correlation coefficient of *R*^2^ = 0.9998 (Figure S2). For the control group (CK), two peaks were observed with the main peak occurring at a retention time of 18.67 min, corresponding to an Mw of 2576 Da that accounted for 62.39% of the total area (Figure 3a and Table S2). Additionally, another peak with an Mw of 29,340 Da that accounted for 37.61% of total area was detected at 17.68 min. In comparison, the Se(IV) treatment group exhibited a main peak at 18.68 min, with an Mw of 2741 Da, accounting for 62.57% of the total area (Figure 3a and Table S2). Meanwhile, another peak with an Mw of 31,976 Da that accounted for 37.43% of the total area was detected at 17.68 min. The Mw distribution was similar to that of the control group with the Mw slightly increased, suggesting that Se(IV) treatment had a limited effect on polymerization [32]. For the Se(VI) treatment group, the main peak appeared at 18.46 min with an Mw of 3705 Da, accounting for 82.71% of the total area (Figure 3a and Table S2). This shift toward the proportion of low-Mw polysaccharides suggested that Se(VI) treatment would result in a more concentrated polysaccharide composition with a lower Mw. In addition, a dominant peak area (90.9%) was exhibited in the SeOP group at 19.37 min with the detected Mw of 4830 Da, with less polysaccharides of a high Mw (1,332,040) observed (Figure 3b and Table S2). Similarly, the PSeCA sample exhibited a low Mw peak (5086 Da) accounting for 91.6% of the total area (Figure 3b and Table S2). The increased proportion of small Mw observed in Se(VI), PSeCA and SeOP suggested that exogenous Se species might influence Mw distribution of polysaccharides by enzymatic regulation or Se-induced structural modification during polysaccharide biosynthesis. A similar phenomenon has been reported in *P. eryngii* biofortification, with the relative Mw of the polysaccharides decreasing following Se treatments [9]. The small Mw might also be caused by depolymerization during the extraction process. As reported elsewhere, polysaccharides are prone to depolymerization under prolonged exposure to high temperatures or strong acidic conditions [4,9].

### 3.4. FT-IR and NMR Analysis

**Figure 4:**
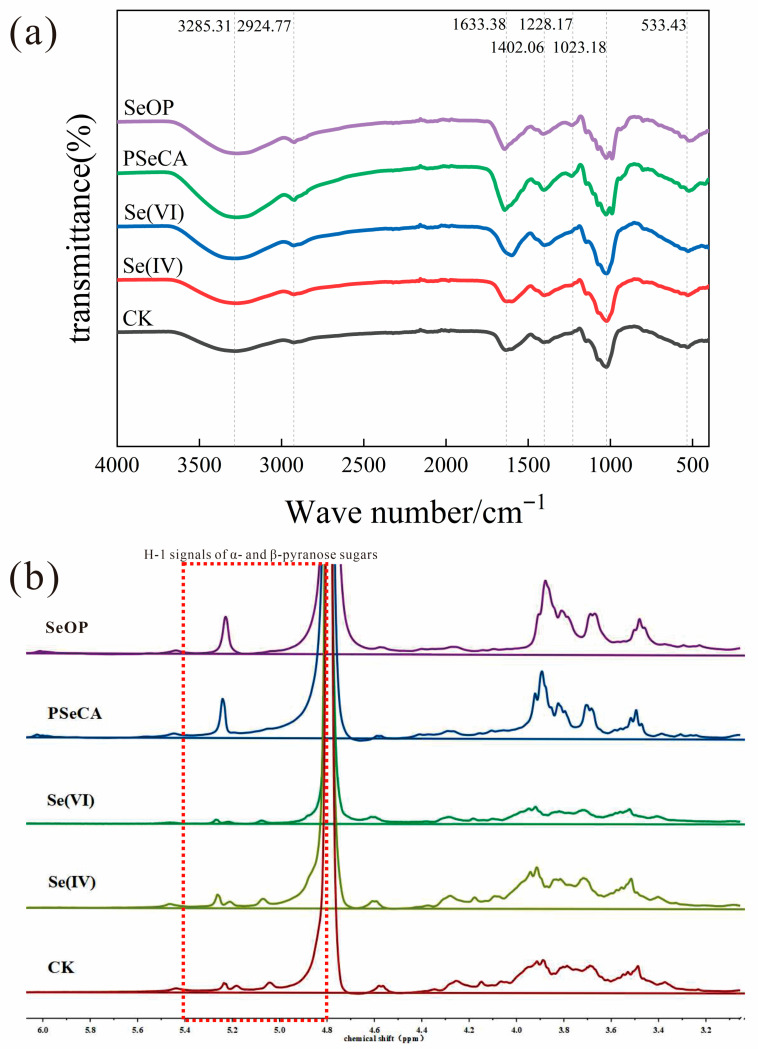
FT-IR (**a**), and ^1^H-NMR (**b**) spectrum of *P. geesteranus* polysaccharides.

The structural differences in the *P. geesteranus* polysaccharides were further evaluated by FT-IR and NMR analysis. As shown in Figure 4a, the FT-IR spectra revealed that major characteristic peaks of all samples were consistent, indicating similar backbone structures among these polysaccharide samples. Nevertheless, variations in peak intensities and shapes were observed, which suggested that Se incorporation could influence the chemical environment and intensity of functional groups. In the high-wavenumber region, the broad absorption band at 3285.31 cm^−1^, corresponding to the stretching vibration of hydroxyl groups (–OH), is a common feature for polysaccharides, indicating the presence of abundant hydroxyl structures [19]. Se-enriched samples (especially for SeOP and PSeCA) exhibited notably stronger peak intensity compared to the control, suggesting Se incorporation might enhance intermolecular hydrogen bonding [4]. The absorption peak at 2924.77 cm^−1^ that was attributed to the stretching vibration of aliphatic C–H was relatively unchanged across all groups, which implied Se treatments could cause less impact on the stability of the aliphatic structures. The peak at 1633.38 cm^−1^ is associated with the stretching vibration of the C=O bond in carboxyl or amide groups, and could also be related to the O–H bending vibration of bound water [33]. The peak at 1402.06 cm^−1^ corresponds to C–H bending or the symmetric stretching vibration of carboxylates (–COO^−^), reflecting the presence of acidic groups in the polysaccharide structure [19]. All Se-fortified samples displayed a stronger absorption in this region as compared to the control group (Figure 4a). The FT-IR shifts suggest possible interactions between Se and carboxyl groups, though the exact chemical bonding requires speciation analysis. The absorption in the fingerprint region (1228.17, 1147.61, and 1023.18 cm^−1^) was mainly attributed to C–O–C, C–OH, and pyranose-type polysaccharide stretching vibrations, which are characteristics of polysaccharide structures [19,20]. The peak at 1023.18 cm^−1^ was particularly prominent, confirming the integrity of the polysaccharide backbone. In the SeOP and PSeCA groups, the intensity of this peak was enhanced, suggesting that Se biofortification may increase the symmetry of the sugar chains or the degree of hydroxyl substitution [16]. In the low-wavenumber region, the peak at 533.43 cm^−1^ was probably attributed to the bending vibration of Se–O or Se–C bonds (Figure 4a). This peak appeared in the Se(IV), Se(VI), PSeCA, and SeOP groups but was absent in the control, confirming the effective doping capacity of Se into the polysaccharide structure [8,34]. However, FT-IR alone was not enough to confirm Se incorporation or the bonding type of polysaccharides. Based on the significantly high (*p* < 0.05) detected Se content of polysaccharides (Table 3), the FT-IR result suggested that Se incorporation might exist in polysaccharides, and other complementary techniques (e.g., XPS, ICP-MS speciation, or XANES) should be further employed to confirm the Se incorporation.

The ^1^H-NMR spectra revealed typical polysaccharide hydrogen spectral features within the range of 6.0–3.2 ppm for all samples (Figure 4b). Specifically, in the 4.8–5.4 ppm region, 1–2 moderate-intensity peaks were observed, corresponding to the H-1 signals of α- and β-pyranose sugars, indicating the presence of multiple sugar residues and a relatively complex main chain structure in all samples [35]. In the 3.2–4.6 ppm region, the H-1 peaks of SeOP and PSeCA groups were more clearly split, suggesting that the sugar chain structure differed from CK (Figure 4b). In PSeCA and SeOP fortification, the increased complexity of the spectra indicated that Se substitution and incorporation could lead to structural changes in the polysaccharide. In contrast, the Se(IV) and Se(VI) groups exhibited rather similar signal features to the control, while the signal intensity greatly changed (Figure 4b). The ^13^C-NMR spectra also exhibited great differences among the four polysaccharides (Figure S3). In the 90–95 ppm region, a C-1 peak (pyranose C-1) was revealed for PSeCA and SeOP samples (Figure S3), which confirmed the presence of pyranose-type chains [34]. In the 60–85 ppm region, PSeCA and SeOP samples were well-resolved and split (Figure S3), probably due to their high purity (Table S2) after chromatography separation. For Se(IV), Se(VI), and CK samples, no distinct signal was observed, likely due to insufficient purity (Table S2) or concentration that could cause signal dispersion [36].

### 3.5. SEM Analysis of Different Polysaccharide Samples

**Figure 5:**
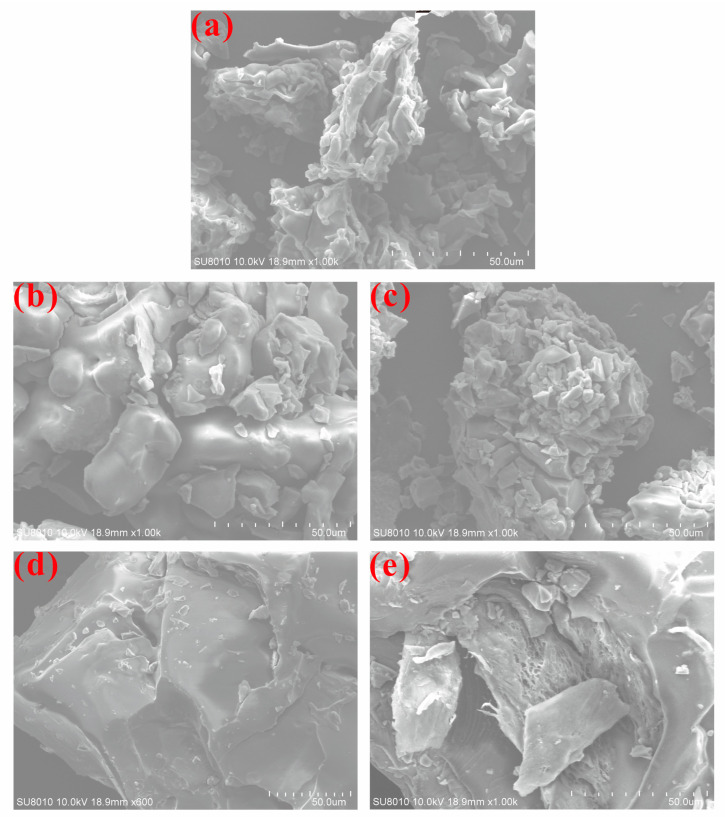
SEM images of *P. geesteranus* polysaccharides under different treatments. (**a**) CK (control), magnified at 1000×; (**b**) Se(IV) treatment, magnified at 1000×; (**c**) Se(VI) treatment, magnified at 1000×; (**d**) PSeCA treatment, magnified at 600×; (**e**) SeOP treatment, magnified at 1000×.

SEM analysis revealed pronounced differences in surface morphology, particle size distribution, and aggregation patterns among the polysaccharide samples (Figure 5 and Figure S4), indicating that different Se sources could play a pivotal role in modulating the spatial conformation of the polysaccharides [37]. The control (CK) group exhibited a loose, fragmented surface with small particles and irregular edges, resulting in a porous and relatively disordered structure (Figure 5). Compared to CK, Se(IV) and Se(VI) samples displayed more compact, blocky, or lamellar structures with tightly bound particles, and some areas formed thin-film-like stacks (Figure 5). The PSeCA sample displayed large, layered block structures that were relatively smooth, with localized edges showing some fracturing and cracking (Figure 5). This demonstrated that PSeCA biofortification might influence the stacking of the polysaccharide skeleton differently to Se(IV) or Se(VI). In addition, SeOP exhibited significantly different features, with rough, porous surfaces and loosely packed lamellar structures (Figure 5). Furthermore, the PSeCA group formed large blocky layered structures, while the SeOP group exhibited rough and porous characteristics (Figure 5).

### 3.6. Antioxidant Activity of P. geesteranus Polysaccharides

**Figure 6:**
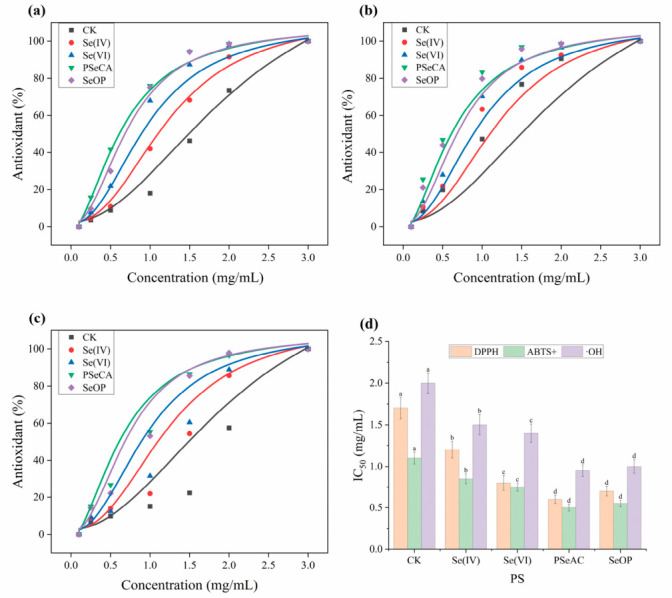
Antioxidant activities of different *P. geesteranus* polysaccharides. (**a**) DPPH radical scavenging activity curves; (**b**) ABTS^+^ radical scavenging activity curves; (**c**) OH radical scavenging activity curves; (**d**) IC_50_ values of DPPH, ABTS^+^, and ·OH radical scavenging activities. Different letters within the same assay indicate significant differences (*p* < 0.05).

The antioxidant assay indicated that Se biofortification significantly enhanced the radical scavenging abilities of the polysaccharides, with Se-enriched polysaccharides showing a marked improvement in DPPH, ABTS^+^, and ·OH scavenging activities compared to the control (CK) group (Figure 6a–c). The GraphPad Prism 9.1.0 software was used to calculate IC_50_ values of each sample. The IC_50_ values of CK were found to be 1.7 mg/mL for DPPH, 1.1 mg/mL for ABTS^+^, and 2.0 mg/mL for ·OH. In contrast, Se(VI)-fortified mushroom polysaccharides exhibited lower IC_50_ values of 1.0 mg/mL for DPPH, 0.75 mg/mL for ABTS^+^, and 1.4 mg/mL for ·OH (Figure 6d). This is consistent with a previous study that showed Se-containing polysaccharides could enhance electron donation or hydrogen-atom transfer capabilities [38]. The antioxidant activity of the PSeCA sample was the most pronounced, with IC_50_ values of 0.6 mg/mL for DPPH, 0.50 mg/mL for ABTS^+^, and 0.95 mg/mL for ·OH (Figure 6d). The enhanced effect might be attributed to the introduction of organic Se groups (Se–C≡N) into the polysaccharide backbone, which altered the glycosidic configuration and increased the number of electron-rich functional groups and the branching degree of the molecules. Previous studies have shown that increased branching, higher carboxyl group content, and a moderate increase in the Mw of Se-enriched polysaccharides were all significantly associated with improved antioxidant capacity [39]. As a slow-release Se source, SeOP treatment contributed to moderate enhancement of antioxidant properties in mushroom polysaccharides when compared with the control, with IC_50_ values of 0.7 mg/mL for DPPH, 0.55 mg/mL for ABTS^+^, and 1.0 mg/mL for ·OH (Figure 6d).

**Figure 7:**
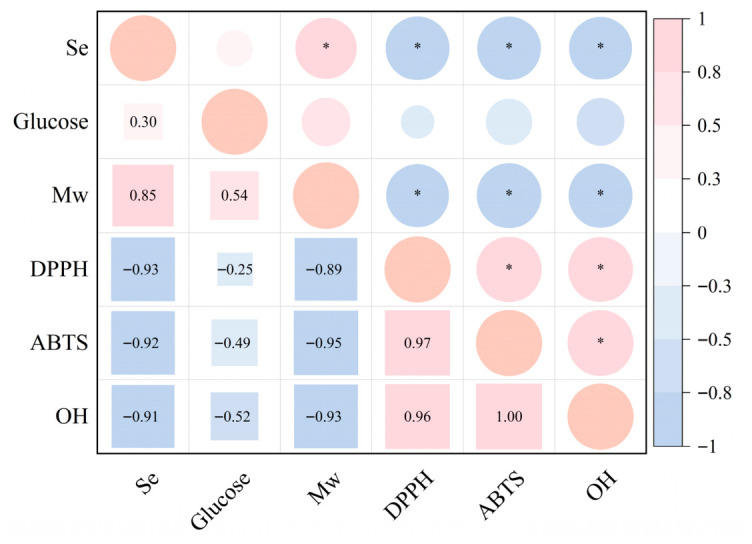
Correlation analysis between selenium content, glucose content, and antioxidant activity. Glucose is expressed as percentage content, and antioxidant activity is represented by the IC_50_ value, and * refers *p* < 0.05.

Additionally, correlation between Se content (Table 3), glucose content (Table S1), and antioxidant activity (Figure 6) were evaluated using the multivariate Pearson correlation analysis method [17]. As shown in Figure 7, the results suggested that the Se content of polysaccharides revealed negative correlation with the IC_50_ values of tested free radicals, with a coefficient value of −0.93, −0.92, and −0.91 observed for DPPH, ABTS, and ·OH radical scavenging respectively. This suggested Se content was positively correlated with the antioxidant capacities of polysaccharides, and confirmed that the four Se species exhibited various capacity to enhance the antioxidant bioactivity of polysaccharides. The antioxidant activity of polysaccharides is closely associated with their hydrogen-donating capacity, and the presence of Se in polysaccharides could activate the hydrogen atom of the anomeric carbon [4,9]. A similar result was also observed between the Mw and antioxidant capacity of polysaccharides, which showed significant negative correlations (ranging from −0.89 to −0.93) with IC_50_ values of tested free radicals, suggesting that polysaccharides with a low Mw may possess stronger antioxidant activity (Figure 7). As reported elsewhere, low-Mw polysaccharides of fungi usually possess higher bioactivity and bioavailability compared to high-Mw polysaccharides [40]. In addition, glucose content (of polysaccharides) exhibited a moderate positive correlation with molecular weight (r = 0.54), and its correlations with antioxidant activities were relatively weak (ranging from −0.25 to −0.52) (Figure 7). Based on the results of Figure 6 and Figure 7, PSeCA and SeOP revealed great potential in the production of bioactive Se-polysaccharides due to high antioxidant properties among the four samples. In the future, the in vivo bioactivity and toxicity of these polysaccharides should be further explored for better utilization of fungi-derived Se-polysaccharides.

## 4. Conclusions

In this study, four different biofortification methods were used to obtain Se-enriched polysaccharides from *P. geesteranus*. The extraction of polysaccharides was optimized using UAE combined with RSM, with the optimal parameters of ultrasonic power 320 W, extraction time 31 min, temperature 77 °C, a liquid-to-solid ratio of 21 mL/g and maximum extraction yield of 70.26 mg/g being observed. Se biofortification revealed that PSeCA exhibited the highest extraction yield (75.12 mg/g). Nevertheless, the monosaccharide composition and the Mw of polysaccharides could be changed under various Se treatments. Further, the FT-IR, NMR, and SEM analysis indicated that Se biofortification altered the structural characteristics of *P. geesteranus* polysaccharides, indicating that Se treatment might actively participate in polysaccharide biosynthesis and modulate macromolecular assembly. The highest polysaccharide yield, Se content (13.85 mg/kg), and antioxidant capacity achieved by PSeCA provided a direct recommendation for seeking an efficient biofortification strategy. However, a lack of in vivo validation, absence of toxicity evaluation, and limited structural elucidation of polysaccharides greatly restricts its future applicability. Future studies are still needed to elucidate the precise structure of Se-enriched polysaccharides as well as their biological efficacy in vivo to develop functional food ingredients and therapeutic biomolecules.

## Figures and Tables

**Table 1:** Results of response surface analysis for polysaccharide extraction yield.

Run	(A)/W	(B)/min	(C)/°C	(D)/mL·g^−1^	Extraction Yield (mg/g)	Predicted Yield (mg/g)
1	200	20	80	20	52.32	52.65
2	400	20	80	20	57.84	65.02
3	200	40	80	20	55.37	38.88
4	400	40	80	20	59.52	55.42
5	300	30	70	15	61.33	61.47
6	300	30	90	15	51.41	61.79
7	300	30	70	25	62.84	63.15
8	300	30	90	25	52.43	66.71
9	200	30	80	15	53.75	45.18
10	400	30	80	15	60.64	63.95
11	200	30	80	25	56.33	48.23
12	400	30	80	25	62.19	67.50
13	300	20	70	20	60.57	59.99
14	300	40	70	20	62.46	53.62
15	300	20	90	20	49.27	62.69
16	300	40	90	20	51.26	54.82
17	200	30	70	20	52.17	44.37
18	400	30	70	20	58.26	64.23
19	200	30	90	20	46.39	47.15
20	400	30	90	20	49.63	65.33
21	300	20	80	15	61.28	60.20
22	300	40	80	15	62.67	53.94
23	300	20	80	25	63.03	64.36
24	300	40	80	25	64.51	56.38
25	300	30	80	20	68.43	70.74
26	300	30	80	20	69.26	70.74
27	300	30	80	20	68.59	70.74
28	300	30	80	20	69.81	70.74
29	300	30	80	20	68.62	70.74

(A) Ultrasonic power, (B) extraction time, (C) extraction temperature, (D) liquid-to-solid ratio.

**Table 2:** Results of ANOVA for the polysaccharide extraction quadratic model.

Source	Sum of Squares	df	Mean Square	F-Value	*p*-Value	
Model	1237.51	14	88.39	87.04	<0.0001	***
A	84.01	1	84.01	82.72	<0.0001	***
B	10.98	1	10.98	10.81	0.0054	**
C	273.03	1	273.03	268.85	<0.0001	***
D	8.76	1	8.76	8.62	0.0108	*
AB	0.4692	1	0.4692	0.4620	0.5078	
AC	2.03	1	2.03	2.00	0.1792	
AD	0.2652	1	0.2652	0.2612	0.6173	
BC	0.0025	1	0.0025	0.0025	0.9611	
BD	0.0020	1	0.0020	0.0020	0.9650	
CD	0.0600	1	0.0600	0.0591	0.8114	
A^2^	457.43	1	457.43	450.43	<0.0001	***
B^2^	100.51	1	100.51	98.97	<0.0001	***
C^2^	548.59	1	548.59	540.19	<0.0001	***
D^2^	37.29	1	37.29	36.72	<0.0001	***
Residual	14.22	14	1.02			
Lack of Fit	12.87	10	1.29	3.83	0.1037	
Pure Error	1.34	4	0.3361			
Cor Total	1251.72	28				
*R*^2^ = 0.9886		*CV* = 1.71%			*R*_Adj_^2^ = 0.9773	

(A) Ultrasonic power, (B) extraction time, (C) extraction temperature, (D) liquid-to-solid ratio. * *p* < 0.05, ** *p* < 0.01, and *** *p* < 0.001.

**Table 3:** Extraction yield and Se content of polysaccharides (PS) from *P. geesteranus*.

Group	PS Yield (mg/g)	Se Content of PS (mg/kg)	Se Recovery Rate (%)	Purity of PS (%)
CK	70.26	0.36	0.86	71.27
Se(IV)	71.57	5.57 *	2.79	72.95
Se(VI)	68.34	8.34 *	4.17	68.54
PSeCA	75.12	13.85 *	6.93	72.35
SeOP	70.93	7.93 *	3.97	73.33

* *p* < 0.05 as compared with CK, CK: the control untreated with Se species in cultivation.

## Data Availability

The original contributions presented in this study are included in the article/Supplementary Materials. Further inquiries can be directed to the corresponding author.

## Supplementary Materials

The following supporting information can be downloaded at: https://www.mdpi.com/article/10.3390/foods15101660/s1, Table S1: Monosaccharide composition of *P. geesteranus* polysaccharides; Table S2: Mw distribution of different *P. geesteranus* polysaccharides; Table S3: Levels of factors in the response surface experiments; Figure S1: Effect of (a) ultrasonic power, (b) ultrasonic time, (c) ultrasonic temperature, and (d) liquid-to-solid ratio on polysaccharide extraction yield; Figure S2: Molecular weight standard curve; Figure S3: ^13^C-NMR spectrum of *P. geesteranus* polysaccharides; Figure S4: SEM images of *P. geesteranus* polysaccharides under different treatments ((a,b) CK (control), 100× and 250×; (c,d) Se(IV) treatment, 100× and 250×; (e,f) Se(VI) treatment, 100× and 250×; (g,h) PSeCA treatment, 100× and 250×; (i,j) SeOP treatment, 100× and 250×).
