# Supplementary material for: Influence of Different Selenium Biofortification Methods on Structural Features and Antioxidant Bioactivities of Pleurotus geesteranus Polysaccharides
Source: Foods. 2026 May 9;15(10):1660. doi: 10.3390/foods15101660 (PMC13205982; doi:10.3390/foods15101660)
Supplement: Supplementary file 1 [file foods-15-01660-s001.zip › foods-4277031-supplementary.pdf]

Table

Table S1. Monosaccharide composition of *P. geesteranus* polysaccharides.

No.	Monosaccharide	RT (min)	CK (%)	Se(IV) (%)	Se(VI) (%)	PSeCA (%)	SeOP (%)
1	Fucose	2.584	0.75	0.5			
2	Amino-Galactose	4.609	0.18			0.79	0.75
3	Rhamnose	4.934					
4	Arabinose	5.284	0.67	0.24	4.35		
5	Amino-Glucose	5.792	6.19	3.47		1.6	1.35
6	Galactose	6.634	15.9	15.33		4.14	2.51
7	Glucose	7.567	65.25	68.52	38.55	85.82	88.69
8	Xylose	8.842				0.42	
9	Mannose	9.159	9.36	9.72	39.41	4.79	5.38
10	Fructose	10.384		0.46	17.69	1.81	1.32
11	Galacturonic Acid	19.242		1.76		0.64	
12	Glucuronic Acid	20.009	1.7				

CK: The control group that untreated with Se biofortification.

Table S2. Mw distribution of different *P. geesteranus* polysaccharides.

Sample	Peak No.	RT (min)	Mn	Mw	Mp	Area	Area (%)	Se content
CK	1	17.68	18214	29340	8393	313196	37.61	0.29
	2	18.67	1221	2576	2395	519544	62.39	mg/kg
Se(IV)	1	17.20	19592	31976	15463	291736	37.43	6.23
	2	18.68	1265	2741	2361	487752	62.57	mg/kg
Se(VI)	1	17.30	24336	40943	13665	142331	17.29	8.98
	2	18.46	1556	3705	3127	680692	82.71	mg/g
PSeCA	1	13.92	538572	1952977	1009601	65782	8.40	14.55
	2	19.66	1021	5086	676	717766	91.60	mg/kg
SeOP	1	14.02	521092	1332040	889041	71863	9.10	7.91
	2	19.73	902	4830	625	717705	90.90	mg/kg

Table S3. Levels of factors in the response surface experiments.

Level	A	B	C	D
-1	200	20	70	15
0	300	30	80	20
1	400	40	90	25

A: ultrasonic power (W), B: extraction time (min), C: extraction temperature (℃), D: liquid to solid ratio (mL/g).

Figure

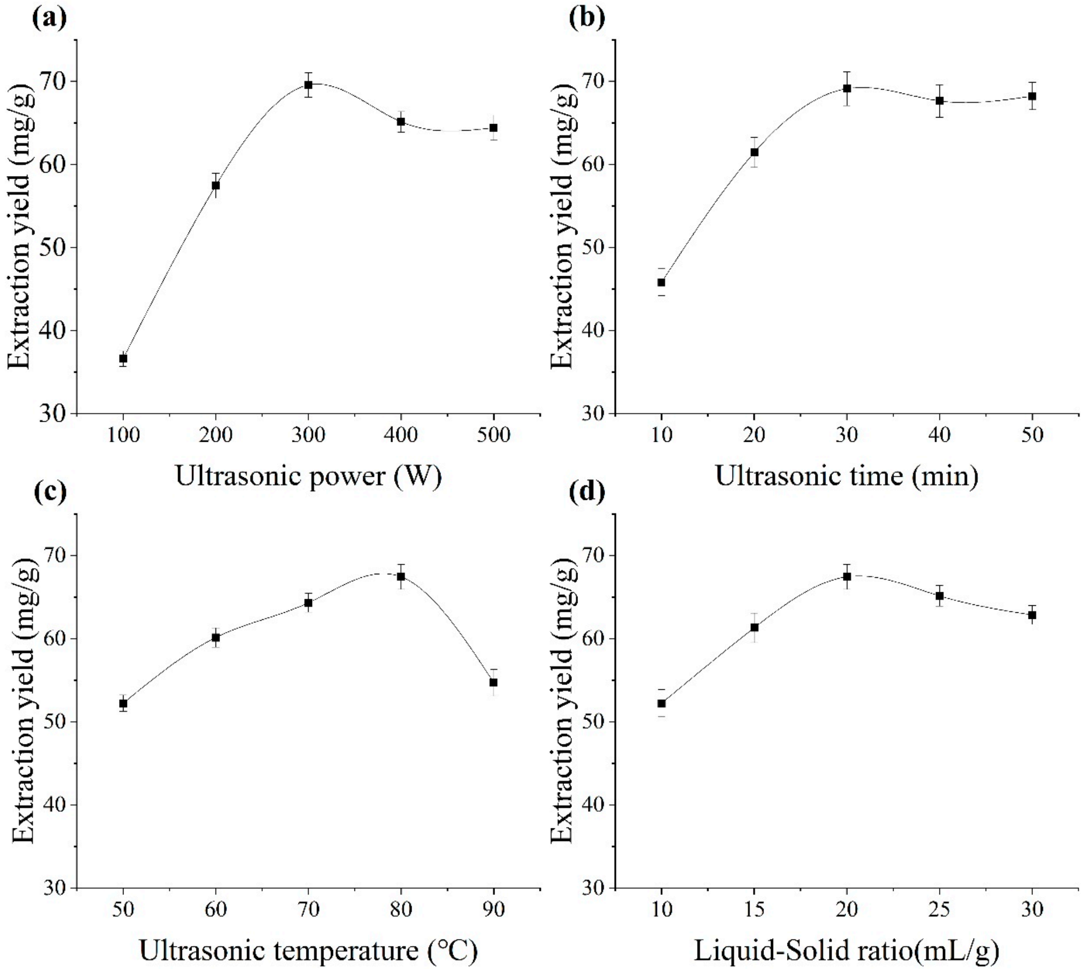

Figure S1. Effect of (a) ultrasonic power, (b) ultrasonic time, (c) ultrasonic temperature, and (d) liquid-to-solid ratio on polysaccharide extraction yield.

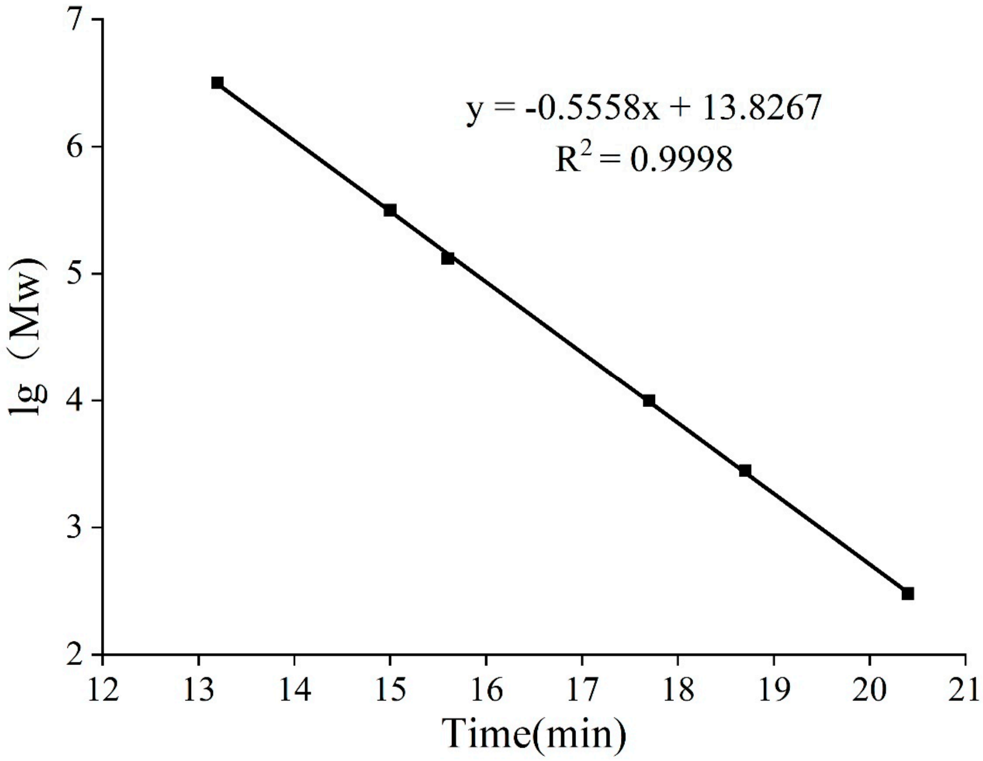

Figure S2. Molecular weight standard curve.

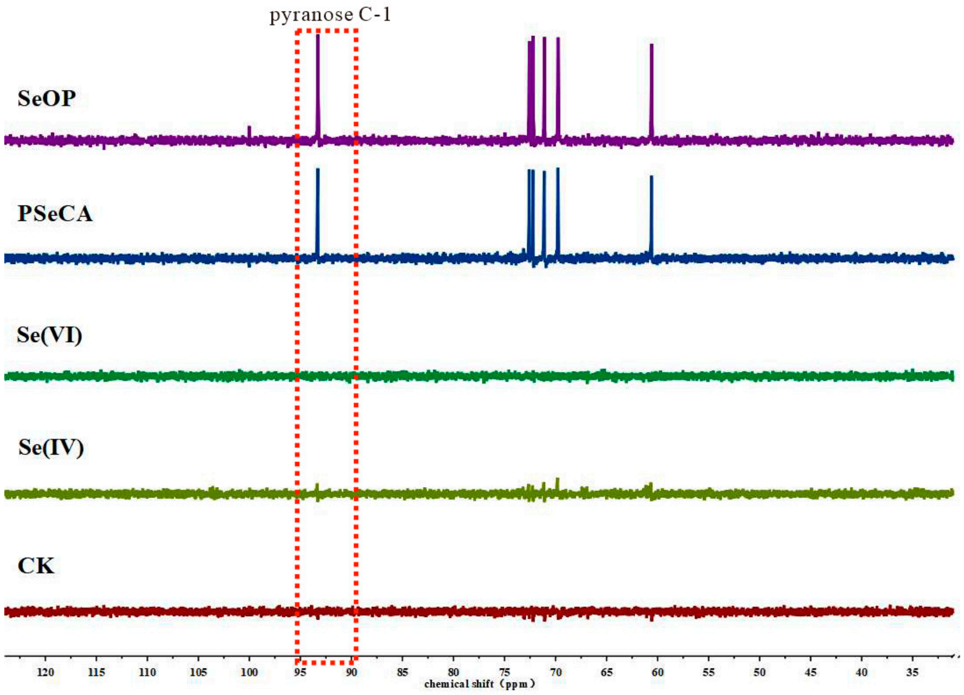

Figure S3. ^{13}C-NMR spectrum of *P. geesteranus* polysaccharides.

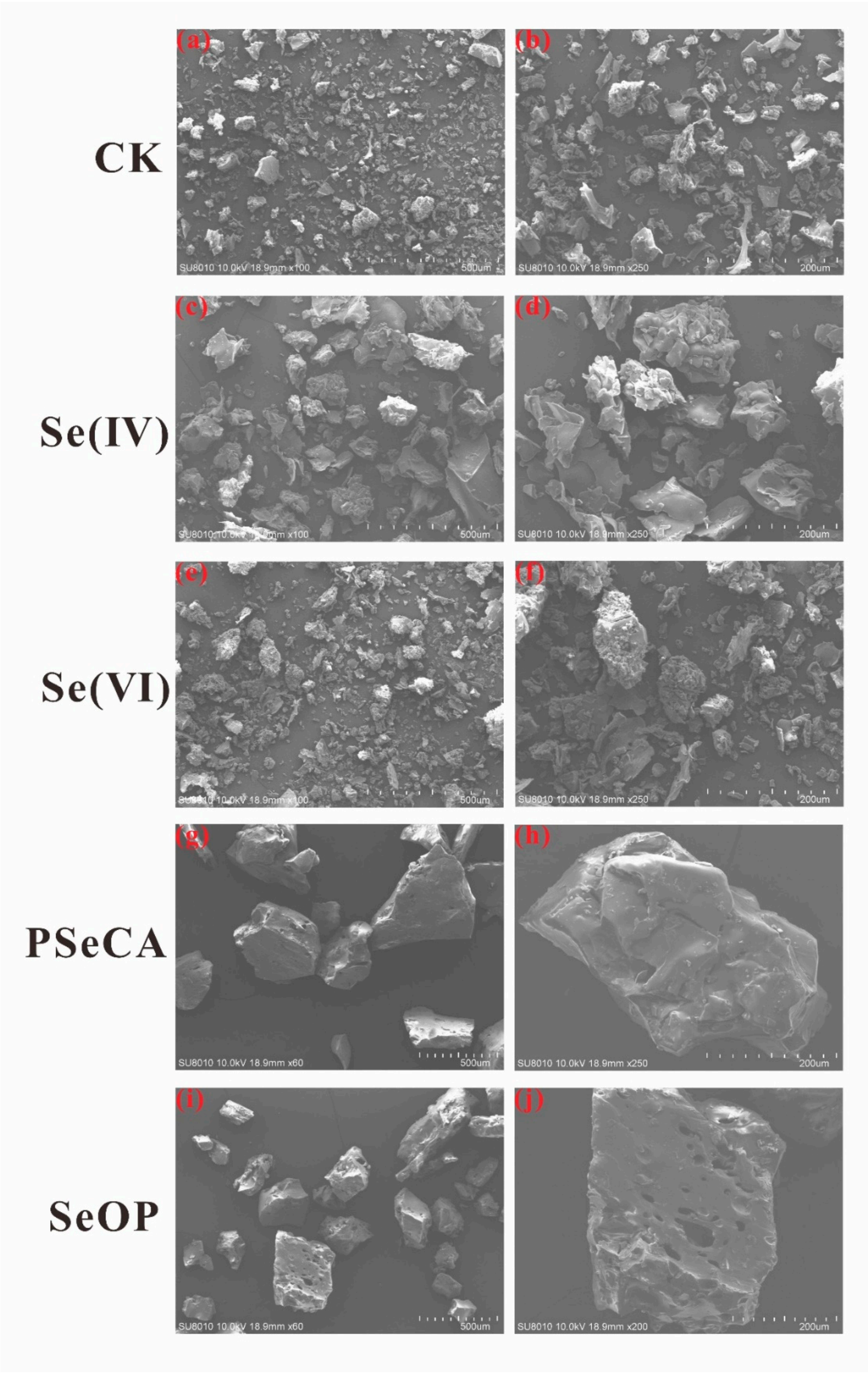

Figure S4. SEM images of *P. geesteranus* polysaccharides under different treatments. (a-b) CK (control), magnified at 100×, and 250×; (c-d) Se(IV) treatment, magnified at 100×, and 250×; (e-f) Se(VI) treatment, magnified at 100×, and 250×; (g-h) PSeCA treatment, magnified at 100×, and 250×; (i-j) SeOP treatment, magnified at 100×, and 250×.
